# Development of auditory scene analysis: a mini-review

**DOI:** 10.3389/fnhum.2024.1352247

**Published:** 2024-03-12

**Authors:** Axelle Calcus

**Affiliations:** Center for Research in Cognitive Neuroscience (CRCN), ULB Neuroscience Institute (UNI), Université Libre de Bruxelles, Brussels, Belgium

**Keywords:** development, auditory scene analysis (ASA), streaming, selective attention, neurophysiology

## Abstract

Most auditory environments contain multiple sound waves that are mixed before reaching the ears. In such situations, listeners must disentangle individual sounds from the mixture, performing the auditory scene analysis. Analyzing complex auditory scenes relies on listeners ability to segregate acoustic events into different streams, and to selectively attend to the stream of interest. Both segregation and selective attention are known to be challenging for adults with normal hearing, and seem to be even more difficult for children. Here, we review the recent literature on the development of auditory scene analysis, presenting behavioral and neurophysiological results. In short, cognitive and neural mechanisms supporting stream segregation are functional from birth but keep developing until adolescence. Similarly, from 6 months of age, infants can orient their attention toward a target in the presence of distractors. However, selective auditory attention in the presence of interfering streams only reaches maturity in late childhood at the earliest. Methodological limitations are discussed, and a new paradigm is proposed to clarify the relationship between auditory scene analysis and speech perception in noise throughout development.

## 1 Introduction

Contrary to appearances, lively playgrounds and business meetings have one thing in common: they are noisy. In such complex auditory environments, sound waves are mixed before reaching the ears. Listeners must disentangle individual sounds from the mixture, performing what is called the *auditory scene analysis* (ASA; Bregman, 1990, 2015). Analyzing complex auditory scenes relies on the listeners' ability to segregate acoustic events into different streams, and to selectively attend to the stream of interest.

**Figure 1:**
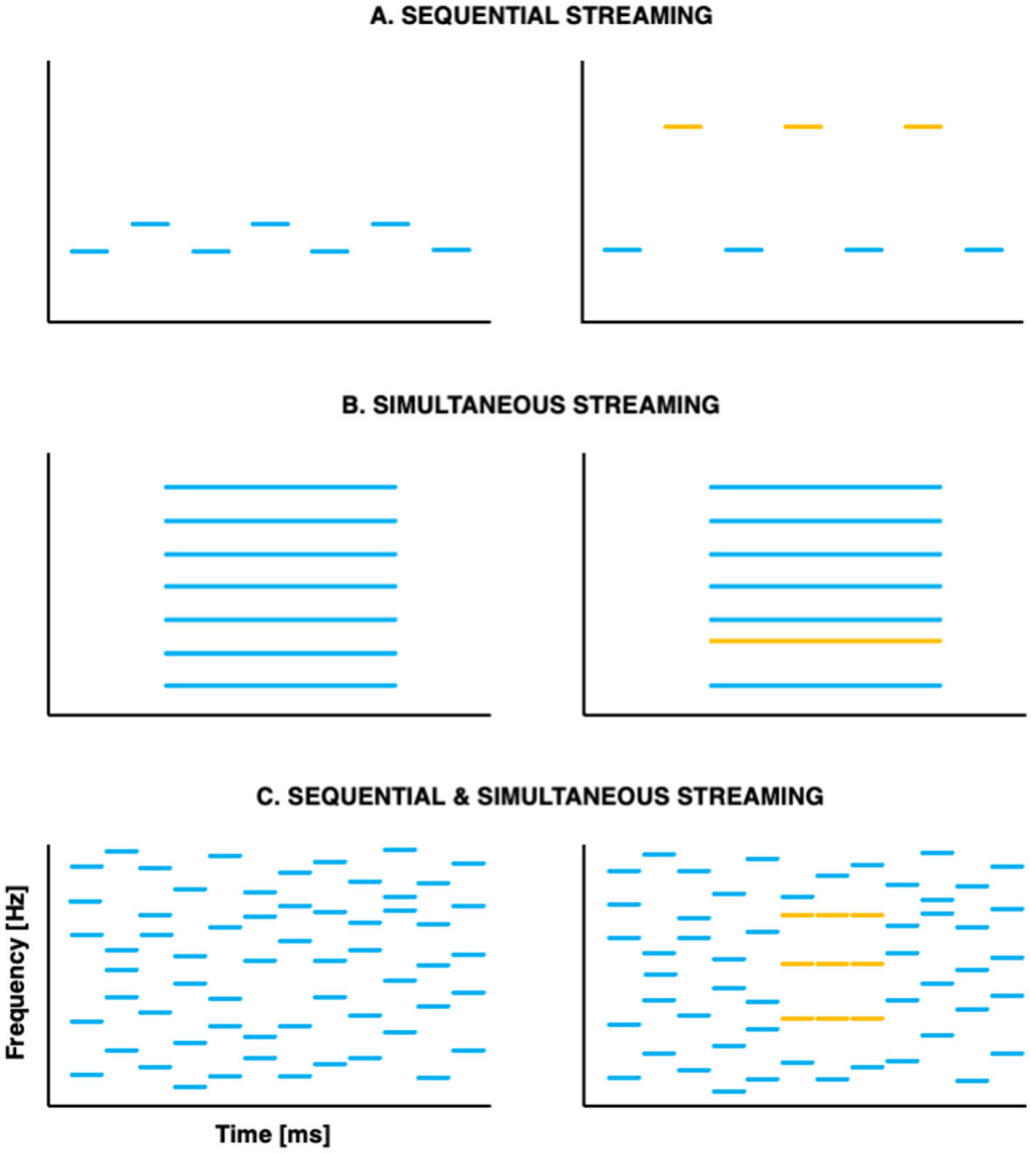
Schematic representation of the canonical paradigms used to investigate stream segregation **(A)** in sequences of successive tones; **(B)** in simultaneous concurrent sounds and **(C)** in stochastic tone clouds that combine successive and simultaneous tones. Stimuli that are typically perceived as one auditory stream are shown on the left; stimuli that are typically perceived as two auditory streams are shown on the right—with the two streams shown as different colors.

With respect to segregation, pioneer studies used sequences of tones organized temporally in repeated ABABAB patterns, where A and B represent successive tones of different frequencies (e.g., Miller and Heise, 1950; see Figure 1A). When listeners report hearing two streams, they are effectively experiencing stream segregation: they parse the *sequential* auditory events into distinct streams. At a given presentation rate, the larger the frequency distance between A and B, the more likely participants are to experience stream segregation. Later studies set out to evaluate segregation abilities in response to *simultaneous*, concurrent sounds. Listeners were presented with complex harmonic tones, of which one component had been mistuned (Moore et al., 1986; see Figure 1B) or delayed (Hedrick and Madix, 2009); manipulations that contributed to segregation into distinct auditory objects. With respect to selective attention, canonical studies investigated adults' ability to focus on a specific auditory feature in the presence of simultaneous or sequential distractors (e.g., Greenberg and Larkin, 1968).

A major limitation of these early studies is their focus on either sequential or simultaneous stimuli. However, in everyday life, broadband streams that are temporally correlated often overlap with one another. In such situations, temporal coherence between different elements of the auditory scene appears essential for auditory segregation (Elhilali et al., 2009), potentially guiding selective attention such that it binds together coherent acoustic (spectral, spatial, and/or temporal) features into streams (Shamma et al., 2011). In this view, attention contributes not only to stream selection, but also to stream formation. An interesting development of the past decade was the creation of a paradigm in which the spectral coherence varies across time, requiring listeners to perform *both* simultaneous and sequential streaming at once (Teki et al., 2011, 2013; see Figure 1C).

How ASA develops in the first decades of life has attracted a lot of interest over the years. So far, studies focused on paradigms that tackled either sequential or simultaneous ASA. In a comprehensive review published about a decade ago on the topic, Leibold (2011) showed that sequential stream segregation and selective attention are functional early in life, albeit not yet as efficient as they are in adulthood. At the time, the author identified several open questions regarding the development of ASA: (i) How does simultaneous ASA develop from infancy to adulthood? (ii) Which acoustic cues are used by infants/children to perform ASA? (iii) How does sensorineural hearing loss affect the development of ASA? Here, we aim to review recent developmental data that answer some of these questions or raise new interrogations. We focus on studies using non-linguistic stimuli, to illustrate the development of basic auditory perception and processing involved in ASA, without the confound of language abilities.

## 2 Stream segregation

### 2.1 First year of life

Pioneer studies of ASA development investigated sequential streaming in the 1st year of life by habituating infants to a repeating (forward) sound sequence, then measuring their dishabituation to a reversed version of the sequence. Should infants parse the auditory scene based on each individual sound of the sequence, they would show a dishabituation response to the reversed pattern. On the contrary, newborns and 3-month-olds appeared to parse the streams of complex auditory scenes using the same cues adults use (Demany, 1982; McAdams and Bertoncini, 1997; Smith and Trainor, 2011)—albeit less accurately (for a detailed review, see Leibold, 2011).

Later studies investigated the neural correlates of sequential segregation in infants, using the mismatch negativity (MMN). The brain generates a MMN when it processes a difference between an unexpected auditory stimulus (a deviant) and the neural representation of a standard, expected pattern (for a review, see Näätänen et al., 2012). In adults, this “oddball paradigm” would even entail an MMN in the presence of interleaved sounds of a different frequency, as long as the interleaved sounds are perceived as separate streams (Sussman et al., 1999). Presented with this “interleaved” oddball paradigm, newborns also show an MMN, indicating that the neural correlates of sequential stream segregation are functional from birth (Winkler et al., 2003). Seven-month-olds also show an MMN if the deviant is placed in a chord component, and successive chords are played as a sequence (Marie and Trainor, 2013). Note that in this case, infants, like adults (Fujioka et al., 2005), show larger MMN to a deviant in the high than low voice, supporting early emergence of a preference for the highest stream.

In the last decade, a number of studies have set out to investigate the early development of *simultaneous* ASA, answering one of the open question identified by Leibold (2011). Folland et al. (2012) presented 6-month-old infants with complex tones consisting of 6 harmonic components. In half of the trials, one of the harmonic components was mistuned by 2–8% of its initial value. Infants were able to discriminate 4% mistuning or larger, whereas adults' thresholds were between 1 and 2% mistuning. Smith et al. (2017) paired in-tune and 8% mistuned complex tones with visual displays showing either one or two bouncing balls, hence being congruent or incongruent with the complex tones. Four-month-olds looked longer at incongruent audiovisual displays, indicating that they use harmonicity as a cue for stream segregation when integrating multisensory information. Whether newborns can segregate simultaneous auditory objects, or use acoustic cues to guide simultaneous streaming remains an open question.

To our knowledge, only two studies have investigated the neural correlates of simultaneous segregation in the 1st year of life, leading to contradictory results. Both studies used a similar paradigm, where half of the trials were 500 ms long complex tones of which the second harmonic was mistuned by 8% of its original value while the other half were in-tune complex tones. The object-related negativity (ORN) is an event-related potential that indexes listeners' processing of two simultaneous auditory objects (Alain et al., 2001). It is typically elicited by a mistuned component in otherwise harmonic complex tones (see Figure 1B). Whereas, newborns (Bendixen et al., 2015) and 4- to 12-month-old infants (Folland et al., 2015) showed an ORN in response to the mistuned complex tones, 2-month-olds did not (Folland et al., 2015). Future studies are needed to determine whether this discrepancy is due to methodological differences between the studies, or whether they reflect non-linearities in the development of the neural correlates of simultaneous stream segregation.

### 2.2 Childhood

For the sake of this review, childhood will be defined as ranging from 3 to 12 years of age. Most behavioral studies of stream segregation in children have been reviewed in Leibold (2011). They show that the acoustic difference required to segregate sequential or simultaneous sounds into distinct streams decreases as children grow older, but remains larger in late childhood than in adulthood (Alain et al., 2003; Sussman et al., 2007; Sussman and Steinschneider, 2009). Note that 5- to 13- year-old children benefit from visual cues helping simultaneous ASA to the same extent as adults (Bonino et al., 2013). Yet 5-year-olds show less benefit from spatial cues to perform simultaneous stream segregation than adults (Wightman et al., 2003).

Electrophysiological studies are in line with the behavioral observation of immature stream segregation in children up to 12 years of age. Like infants, children show an MMN when presented with stimuli that entail sequential streaming (Sussman et al., 2001; Lepistö et al., 2009). However, the frequency separation between the successive sounds of these sequences needs to be larger in passively attending 9–12 year-olds than adults to elicit an MMN (Sussman and Steinschneider, 2009).

With respect to simultaneous ASA, Alain et al. (2003) recorded the ORN in 8- to 13-year-old children and adults. Their results indicate that children have a *larger* ORN than adults, despite having poorer behavioral performance when segregating streams in the mistuned complex tones. This was interpreted as suggesting greater neuronal activity associated with the perception of separate auditory objects in children than adults. In a recent follow-up to that study, the same team investigated the ORN of 6–12 year-olds with a moderate to severe congenital hearing loss (55–70 dB HL), who were regular hearing aid users (Mehrkian et al., 2022). Note that children with a hearing loss were tested unaided, but sounds were presented at higher sound pressure level than for age-matched children with normal hearing, thus aiming to equate sensation level across groups. Children with a hearing loss had smaller and later ORN than age-matched children with normal hearing. Congenital sensorineural hearing loss thus seem to have a pervasive effect on the central processing of simultaneous streams, that is not merely due to an audibility loss.

### 2.3 Adolescence

In the past decade, researchers started to investigate the maturational trajectory of ASA at adolescence. The frequency separation needed to experience streaming of successive tones did not change between 7 and 15 years (Sussman et al., 2015). However, in the same study, there was a gradual improvement in the ability to detect an intensity deviant in one of two sequential streams. More studies are needed to investigate adolescent development of simultaneous ASA, and to explore the neural correlates of both sequential and simultaneous streaming at adolescence.

## 3 Selective attention in the context of ASA

### 3.1 First year of life

Do infants use selective attention to guide streaming in complex auditory scenes? To address this challenging question, researchers have investigated the effects of non-sensory factors on the detection of an auditory target in the presence of *simultaneous* distractors (for a detailed review, see Leibold, 2011). From 6 months of age, infants rely on temporal (Werner et al., 2009) but not spectral (Werner and Bargones, 1991; Bargones and Werner, 1994) expectations to selectively direct their attention toward a target in the presence of a simultaneous interference. Several questions remain open: are newborns able to selectively direct their attention in complex auditory scenes? Are infants able to selectively attend to a target that unfolds over time in a sequential stream? What are the neural correlates of infants' selective attention in the presence of auditory distractors?

### 3.2 Childhood

Behavioral studies of simultaneous ASA in children have been reviewed by Leibold (2011), and suggest a progressive improvement in selective auditory attention throughout the primary school years (Greenberg et al., 1970; Allen and Wightman, 1995; Stellmack et al., 1997; Leibold and Neff, 2007). A recent psychoacoustic study aimed at understanding the mechanism underlying this progressive improvement (Jones et al., 2015). Reverse correlations were used to estimate which spectral region children and adults paid attention to when asked to detect a 1 kHz target embedded in an unpredictable noise. Results confirmed that 4- to 7-year-olds had poorer thresholds than 8- to 11-year-olds and adults. In fact, younger children were less efficient at analyzing the spectral content of the stimuli than older children. Their poorer thresholds in noise thus likely reflect an inability to selectively attend to the target while ignoring the distractor. How selective attention to sequential sound streams develops during childhood remains so far unexplored.

Neural correlates of selective attention to sequential streams can be investigated using a variation of the “oddball paradigm” described above (Sussman et al., 1999; Winkler et al., 2003). Participants are presented with two streams of interleaved sounds, differing in frequency (see Figure 1A, right panel). They are asked to focus on one of the streams, and to indicate when they detect a deviant within this target stream, while ignoring deviants that appear in the distracting stream. This allows to compare the neural response of the to-be-attended deviant to that of the to-be-ignored deviant, which typically leads to an early frontal positivity followed by a difference negativity (Nd, for a review see Näätänen et al., 2001). Nds were recorded in a group of 9-year-olds, a group of 12-year-olds, and a group of adults (Gomes et al., 2007). Both groups of children exhibited a later Nd than adults, indicating persistent processing immaturities in sequential streaming in late childhood. Whether persistent processing immaturities would also be observed in the neural correlates of simultaneous streaming, despite the seemingly mature behavioral performance (Jones et al., 2015) remains an open question.

### 3.3 Adolescence

Selective auditory attention to a target in the presence of a simultaneous multitone masker seems to be mature by late childhood (Jones et al., 2015). This observation is consistent with earlier results collected in a small cohort of children as well as adolescents and adults (Lutfi et al., 2003). Whereas, 4- to 10-year-old children showed more masking than adults, there was no difference between adolescents (11–16 years) and adults. A principal component analysis was performed on the variance in masking performance, to investigate whether different age groups and/or individuals use different detection strategies. If so, several components would be identified as significantly contributing to the variance observed in masking performance. On the contrary, a single principal component was found to account for more than 80% of variance in masking performance, both across and within age groups. This suggests that children use similar target detection strategies to adults, but that they vary in their selective attention abilities.

A few studies have investigated the neural correlates of selective attention during sequential streaming at adolescence. Nds did not change between 11- and 14 year-olds as they were asked to detect a deviant in a target stream while ignoring those in the distracting stream (D'Angiulli et al., 2008). Interestingly, the early frontal positivity evoked by the to-be-ignored targets was larger in adolescents with poorer executive functioning skills than in those with higher executive function skills (Lackner et al., 2013). Last, an oddball paradigm was presented with different instructions, directing adolescents' attention toward different auditory cues, or away from the auditory modality and toward visual information (Sussman, 2013). The morphology of adolescents' event-related potentials and MMN varied with the instructions, like adults' (Sussman et al., 2002).

Overall, studies did not find developmental effects on the neural correlates of selective attention to sequential streams, which may indicate mature attentional responses at adolescence. Note however that none of the studies reviewed in the above paragraph included a group of adults, which limits interpretation in terms of the maturational trajectory at adolescence. Additionally, how the neural correlates of selective attention in simultaneous segregation tasks develop throughout adolescence remains unexplored.

## 4 Discussion

**Figure 2:**
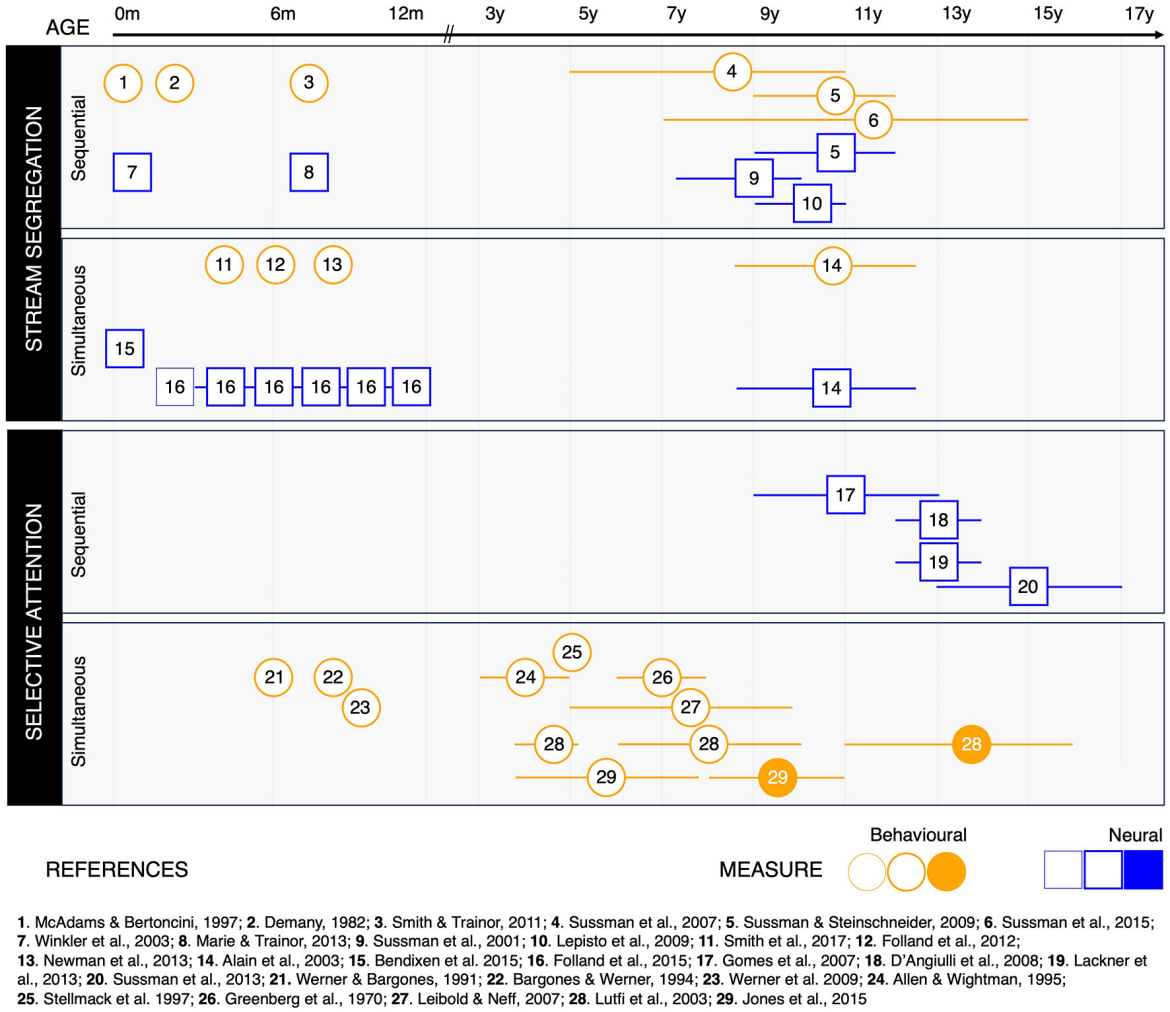
Studies on auditory scene analysis (ASA) throughout development, organized according to the specific ASA ability investigated, the type of measure collected, and the developmental results reported. Behavioral studies are represented as circles (orange); neurophysiological studies are represented as squares (blue). Symbols are positioned at the mean age of the pediatric participants included in the study. Whiskers around the symbols indicate the age range included in the study, whenever relevant. Thin symbols indicate that participants did not show evidence of the ASA ability investigated (Folland et al., 2015). Regular symbols indicate that participants were able to perform ASA. Filled symbols indicate there was no significant difference between the performance of pediatric participants and a group of adults included in the study.

Figure 2 shows the studies reviewed in this paper, with respect to the age range of their pediatric population, the type of measure collected, and the specific ASA ability investigated. To sum up, cognitive and neural mechanisms supporting both simultaneous and sequential stream *segregation* are functional from birth. Yet, their efficiency keeps improving throughout childhood and adolescence (Alain et al., 2003; Sussman et al., 2007, 2015; Sussman and Steinschneider, 2009).

Developmental studies of selective auditory *attention* in the context of ASA paint a seemingly contradictory picture. From 6 months of age, infants benefit from some (but not all) auditory cues to orient their attention toward a target in the presence of simultaneous interferers (Werner and Bargones, 1991; Bargones and Werner, 1994; Werner et al., 2009), in line with neurophysiological data showing developmental changes in arousal over the first 2 years of life (Richards et al., 2010). Yet, the existent developmental data on selective attention in ASA suggest that behavioral performance reaches maturity by late childhood (Lutfi et al., 2003; Jones et al., 2015), whereas its neural correlates keep maturing until adolescence (Gomes et al., 2007). Two explanations might account for this apparent discrepancy. First, children may perform similarly to adults by recruiting different cognitive resources (Trau-Margalit et al., 2023). Future studies are thus warranted to investigate the development of the neural markers of listening effort in noise. Second, the literature on selective attention in the context of ASA seems to present a blind spot. Indeed, to the best of our knowledge, behavioral studies all used simultaneous streaming tasks, whereas neurophysiological studies used sequential streaming tasks. Discrepancies between behavioral and neural results may thus stem from different maturational trajectories between simultaneous and sequential streaming tasks. Note however that speech-in-speech perception inherently requires *both* simultaneous and sequential ASA abilities, whereas the bulk of the literature reviewed here has focused on one or the other. Noteworthily, studies investigating selective attention to speech in the presence of distractors indicate a protracted development of neurophysiological attentional responses from childhood until adulthood (Berman and Friedman, 1995; Karns et al., 2015).

This supports the need to better understand the development of ASA in more ecological situations that require both simultaneous and sequential streaming abilities. The stochastic figure-ground paradigm (Teki et al., 2011, 2013; see Figure 1C) offers a unique opportunity in this respect. The paradigm consists in a series of identical chords (the figure) presented against a background of random chords. Adults are remarkably sensitive to the appearance of such figures in stochastic noise backgrounds—discrimination performance even improves as figure coherence increases. Additionally, the ORN and a later positive wave (P400) have been elicited in adults listening to such stochastic sequences, providing “neural signatures” of figure-ground discrimination (Tóth et al., 2016). Adapting this task to children and adolescents would further our understanding of the development of combined simultaneous and sequential streaming, as is often required in real-life.

Other limitations are that most studies focused on narrow age ranges, and a number of them did not include a group of adult participants. In addition, most of the results reported here stem from single studies that addressed a specific question. In the few cases where more than one study was conducted to address a research question in a specific age range, results were partly contradictory. This points toward the need for comprehensive developmental investigations, including replication studies. This would allow to examine the transition toward adult-like performance, and the factors that contribute to this transition, including those that relate to individual differences in maturation. Cognitive (executive functions and working memory), neurochemical (modulation of serotonin, dopamine and gamma-aminobutyric acid) and environmental factors (exposure to music and language) should be included as potential predictors of maturation, as they are thought to contribute to stream segregation and/or speech perception in noise in adults (Moore et al., 2008; Kondo et al., 2012; Lackner et al., 2013; van Loon et al., 2013; Chabal et al., 2015; Tierney et al., 2020; Porto et al., 2023). Last, future studies are warranted to disentangle the relationship between selective attention and auditory streaming throughout development.

Together, this would pave the way toward a model of ASA development from infancy to adulthood. This would be contribute to understand typical development, and to better grasp the difficulties faced by clinical populations in noisy environments. Many children seem to be disproportionally affected by the presence of background noise (Calcus et al., 2018; Sharma et al., 2019). Adding insult to injury, classrooms are notoriously noisy (Brill et al., 2018). A better understanding of ASA development may therefore have a significant societal impact on the academic performance of children/adolescents in noisy environments.

## Author contributions

AC: Writing—original draft, Writing—review & editing.
